# Compulsive Buying Behavior: Characteristics of Comorbidity with Gambling Disorder

**DOI:** 10.3389/fpsyg.2016.00625

**Published:** 2016-04-29

**Authors:** Roser Granero, Fernando Fernández-Aranda, Trevor Steward, Gemma Mestre-Bach, Marta Baño, Amparo del Pino-Gutiérrez, Laura Moragas, Neus Aymamí, Mónica Gómez-Peña, Núria Mallorquí-Bagué, Salomé Tárrega, José M. Menchón, Susana Jiménez-Murcia

**Affiliations:** ^1^Ciber Fisiopatología Obesidad y Nutrición, Instituto de Salud Carlos IIIBarcelona, Spain; ^2^Departament de Psicobiologia i Metodologia de les Ciències de la Salut, Universitat Autònoma de BarcelonaBarcelona, Spain; ^3^Pathological Gambling Unit, Department of Psychiatry, Bellvitge University Hospital-IDIBELLBarcelona, Spain; ^4^Nursing Department of Mental Health, Public Health, Maternal and Child Health, Nursing School, University of BarcelonaBarcelona, Spain; ^5^Ciber de Salud Mental, Instituto de Salud Carlos IIIBarcelona, Spain

**Keywords:** behavioral addictions, comorbidity, compulsive buying behavior, gambling disorder, prevalence

## Abstract

Compulsive buying behavior (CBB) has begun to be recognized as a condition worthy of attention by clinicians and researchers. Studies on the commonalities between CBB and other behavioral addictions such as gambling disorder (GD) exist in the literature, but additional research is needed to assess the frequency and clinical relevance of the comorbidity of CBB and GD. The aim of the study was to estimate the point-prevalence of CBB+GD in a clinical setting. Data corresponded to *n* = 3221 treatment-seeking patients who met criteria for CBB or GD at a public hospital unit specialized in treating behavioral addictions. Three groups were compared: only-CBB (*n* = 127), only-GD (*n* = 3118) and comorbid CBB+GD (*n* = 24). Prevalence for the co-occurrence of CBB+GD was 0.75%. In the stratum of patients with GD, GD+CBB comorbidity obtained relatively low point prevalence (0.77%), while in the subsample of CBB patients the estimated prevalence of comorbid GD was relatively high (18.9%). CBB+GD comorbidity was characterized by lower prevalence of single patients, higher risk of other behavioral addictions (sex, gaming or internet), older age and age of onset. CBB+GD registered a higher proportion of women compared to only-GD (37.5 vs. 10.0%) but a higher proportion of men compared to only-CBB (62.5 vs. 24.4%). Compared to only-GD patients, the simultaneous presence of CBB+GD was associated with increased psychopathology and dysfunctional levels of harm avoidance. This study provides empirical evidence to better understand CBB, GD and their co-occurrence. Future research should help delineate the processes through which people acquire and develop this comorbidity.

## Introduction

### Compulsive buying disorder and gambling disorder: definition and prevalence

Compulsive buying behavior (CBB) has been described in the psychiatric nomenclature for nearly 100 years, but it attracted little empirical attention until the 1990s when consumer researchers showed the disorder to be widespread (Müller et al., 2015). However, CBB is not included in either the *DSM-5* (American Psychiatric Association, 2013) or the *ICD-10* (World Health Organization, 1992). The currently available operational criteria tend to encompass the cognitive and behavioral aspects of this psychiatric condition (Piquet-Pessôa et al., 2014) and most definitions of CBB agree that this psychiatric conditions is characterized by excessive or poorly controlled urges or behaviors related to shopping and spending, which inadvertently lead to negative consequences (e.g., marked subjective distress, interference in social or occupational functioning and financial/legal problems; Konkolý Thege et al., 2015). Recent research has also outlined that buying tendencies are strongly related with social attitudes toward money, personal finances, and materialistic values. There is also agreement that CBB exists as a separate condition that cannot be attributed to mania or hypomania (Müller et al., 2015). As such, tendencies toward compulsive buying may not necessarily be pathological and CBB should only been seen as representing an extreme form of shopping behavior carried out by the public in general (Spinella et al., 2015).

Epidemiological research evidences that CBB has increased in prevalence in the last years, although little data are currently available regarding accurate point prevalences and validity of the disorder. A recent meta-analysis of 40 studies concluded that the pooled prevalence for CBB in adult representative samples is within the confidence interval of 3.4–6.9%, with higher point estimates for university students (5.9–11.5%), those of non-community origin (7.6–19.1%) and shopping-specific participants (8.8–27.8%; Maraz et al., 2015b). Notwithstanding, these prevalence indexes must be considered with caution, since point estimations in epidemiological research are quite variable, and range from 1 to 30% (Basu et al., 2011). Such results greatly depend upon factors such as the type of sample examined (students, general population or clinical samples), sample sizes, socio-cultural contexts, timeframes (current vs. lifetime) and measurement instruments.

Gambling disorder (GD) has recently been reclassified into the “Substance-Related and Addictive Disorders” group of the DSM-5 (American Psychiatric Association, 2013) and is defined by the presence of persistent and recurrent maladaptive gambling behavior leading to clinically significant impairment or distress. GD is the only behavioral addictive disorder included into this taxonomy as a diagnostic condition.

GD is also increasingly being recognized as a major public health problem. A recent meta-analysis, including 25 studies with overall samples sizes ranging from 80 to 43,093 participants, estimated lifetime prevalence of GD to be within the confidence interval of 0.01–10.6% (Subramaniam et al., 2014). Epidemiological results for age are, however, inconsistent and are strongly related to the origin of the samples and/or the measurement instruments (Erickson et al., 2005).

### Shared characteristics in the CBB and GD phenotype

Evidence for shared features between CBB and GD has been reported, with neurobiological risk factors having received special attention in recent years. As a whole, it seems that shared mechanisms and brain regions are involved in the onset and development of behavioral addictions (i.e., disturbed neurotransmission and alterations in frontoparietal regions, reward processing and limbic systems; Potenza, 2014a; Vanderah and Sandweiss, 2015). Though they tend to use relatively small community samples, the few studies to date assessing gambling behavior in patients with CBB have found these behaviors to be highly associated (Grant and Kim, 2003; Spinella et al., 2015).

The phenotype of CBB and GD also seems to present similar behavioral patterns. Firstly, both psychiatric conditions have been classified as part of the impulse control disorders (ICD) spectrum. Despite some criticisms by clinicians and researchers regarding the validity and real usefulness of this spectrum, the grouping of disorders is of great theoretical interest and could be crucial to enhancing classification schemes. In fact, many of the descriptions of CBB have relied on similarities with disorders in the ICD group (Potenza, 2014b; Robbins and Clark, 2015) and one study found that 95.8% of compulsive buyers described buying behavior that resembled an impulse control disorder (Christenson et al., 1994). Other associations have been found mainly with substance-use disorders (Grant et al., 2013), obsessive-compulsive disorder (Weinstein et al., 2015), eating disorders (Fernández-Aranda et al., 2006, 2008; Jiménez-Murcia et al., 2015), and other behavioral addictions such as gambling disorder (Black et al., 2010).

The most manifest shared attribute of CBB and GD as members of the ICD spectrum is the impulsive/compulsive nature of the behavior in itself. The impulsive/compulsive aspects of buying and gambling behavior have been defined as the failure to resist the impulse, drive or temptation to perform an act, even though it could be harmful to one's own person or to others, in order to achieve immediate gratification or relieve a negative emotion (El-Guebaly et al., 2012; Yi, 2013; Choi et al., 2014; Grant and Chamberlain, 2014; Di Nicola et al., 2015; Konkolý Thege et al., 2015).

Other shared features of CBB and GD are the early onset of the problematic addictive problems, (Balogh et al., 2013; Maraz et al., 2015b), low education levels (Maraz et al., 2015a), high exposure to adverse life events (Lee et al., 2012), and specific personality traits (Black et al., 2012; Di Nicola et al., 2015; Munno et al., 2016). It is also noteworthy that both conditions are characterized by deceit and alterations in attitudes toward money, personal financial behavior, and materialistic values (Black et al., 2010). These conditions also present high levels in positive and negative urgency traits (Rose and Segrist, 2014), familial psychiatric history (Rennert et al., 2014), and comorbid mental health disorders (Mueller et al., 2010a,b; Lorains et al., 2011; Rash et al., 2011; Aboujaoude, 2014; Cowlishaw et al., 2014; Dowling et al., 2015).

### Aims of the study

Empirical data on the frequency of CBB and GD, their phenotypes, risk factors and shared characteristics are available. However, to our knowledge no large study has assessed the clinical co-occurrence of CBB with GD, and therefore the specific phenotype for the CBB+GD comorbidity remains unknown.

The objectives of this study were: (a) to determine the frequency of the comorbid co-occurrence of CBB+GD in a large clinical sample of patients who sought treatment because of a behavioral addiction; and (b) to assess the main differences in the sociodemographic and clinical profiles between comorbid CBB+GD patients and patients who met criteria for only-CBB and only-GD.

## Materials and methods

### Participants

Participants considered for the study were all the patients who were referred to the Pathological Gambling Unit in the Psychiatry Department at Bellvitge University Hospital (Barcelona, Spain) for treatment because of a behavioral addiction (*n* = 3363). These patients were referred to the Unit through general practitioners or via another healthcare professional; some patients were derived from prison health services, though their treatment was not compulsory. Bellvitge University Hospital is a public hospital certified as a tertiary care center for the treatment of addictive behaviors and oversees the treatment of highly complex cases. The catchment area of the hospital includes over two million people in the metropolitan area of a major city. Data recruitment was from January 2005 to August 2015. The patients included in this sample are part of an ongoing project investigating behavioral addictions. Some patients from this sample have been included in other studies examining distinct aspects of behavioral addictions (Moragas et al., 2015; Granero et al., 2016). Exclusion criteria were the presence of an organic mental disorder, an intellectual disability or an active psychotic disorder.

All participants were diagnosed according to DSM-IV criteria (SCID-I; First et al., 1995), and by using specific questionnaires for each disorder. Interviews were conducted by experienced psychologists and psychiatrists with more than 15 years of experience in the field.

Individuals who met criteria for CBB or GD (*n* = 3221) were selected for this study. Other patients not selected met criteria for internet gaming disorder (*n* = 45), internet addiction (*n* = 90), sexual addiction (*n* = 26), and other addictive behaviors (*n* = 16).

The final sample of *n* = 3221 participants was classified in three groups according to their addictive behavior: CBB without GD (named only-CBB in this study; *n* = 103), GD without CBB (named only-GD in this study; *n* = 3094) and comorbid CBB+GD (*n* = 24).

### Measures

#### Evaluation of current and lifetime substance abuse

Patients were assessed using a shortened, structured clinical face-to-face interview modeled after the Structured Clinical Interview for DSM-IV (SCID-I; First et al., 1995), covering the lifetime presence of impulsive behaviors (namely alcohol and drug abuse, comorbid impulse control disorders, namely CB and GD).

#### Symptom checklist-revised (SCL-90-R; Derogatis, 1990)

This self-report, 90-item questionnaire, measured on an ordinal 3-point scale, evaluates a broad range of psychological problems and psychopathological symptoms. It is structured in nine primary symptom-dimensions: somatization, obsession-compulsion, interpersonal sensitivity, depression, anxiety, hostility, phobic anxiety, paranoid ideation and psychoticism. Three global indices are also available: global severity index (GSI, a measure of overall psychological distress), positive symptom distress index (PSDI, a measure of the symptoms intensity), and positive symptom total (PST, which reflects self-reported symptoms). The Spanish adapted version was used in this study (Derogatis, 2002). Cronbach's alpha (α) in the sample of this study was in the good to excellent range (**Table 2** includes α-values for each scale).

#### Temperament and character inventory-revised (TCI-R; Cloninger, 1999)

The TCI-R is a reliable and valid 240-item questionnaire measured on a 5-point Likert-type scale to evaluate personality traits. It is structured in seven primary personality dimensions: four temperamental factors (novelty seeking, harm avoidance, reward dependence, and persistence) and three character dimensions (self-directedness, cooperativeness, and self-transcendence). The Spanish revised version used in this study (Gutiérrez-Zotes et al., 2004) showed adequate internal consistency (Cronbach's alpha α mean value of 0.87). Cronbach's alpha in the sample of this work was in the good to excellent range (α-values are in **Table 2**).

#### Compulsive buying behavior diagnosis

Diagnostic criteria for CBB were determined according the guidelines set by McElroy et al. (1994). These criteria have received wide acceptance in the research community, although their reliability and validity have not yet been determined (Tavares et al., 2008). It should be noted that no formal diagnostic criteria for CBB have been accepted for either the DSM or the ICD-10. At present, it is recommended that CBB be determined via detailed face-to-face interviews which explore “buying attitudes, associated feelings, underlying thoughts, and the extent of preoccupation with buying and shopping” (Müller et al., 2015).

#### Gambling disorder diagnosis

The diagnostic questionnaire for Pathological Gambling according to DSM criteria (Stinchfield, 2003) was used to determine GD diagnosis. This 19-item questionnaire allows for assessing DSM-IV (American Psychiatric Association, 1994) diagnostic criteria for pathological gambling (in the present study called GD). Convergent validity with the SOGS scores in the original version was very good (*r* = 0.77 for representative samples and *r* = 0.75 for gambling treatment groups; Stinchfield, 2003). Internal consistency in the Spanish adaptation used in this study was α = 0.81 for the general population and α = 0.77 for gambling treatment samples (Jiménez-Murcia et al., 2009). In this study, the total number of DSM-5 criteria for GD was analyzed. Cronbach's alpha in the sample was very good (α = 0.81).

#### South Oaks gambling screen (SOGS; Lesieur and Blume, 1987)

This self-report, 20-item, screening questionnaire discriminates between probable pathological, problem and non-problem gamblers. The Spanish validation used in this work showed excellent internal consistency (α = 0.94) and test-retest reliability (*r* = 0.98) (Echeburúa et al., 1994). Consistency in this study sample was adequate (α = 0.76).

#### Alcohol use disorders identification test (AUDIT; Saunders et al., 1993)

This 10-item self-report questionnaire assesses excessive alcohol consumption (levels of consumption, symptoms of dependence and alcohol-related consequences). Internal consistency has been found to be high, and test-retest data have suggested high reliability (0.86) and sensitivity around 0.90; specificity in different settings and for different criteria averages 0.80 or more. Three groups were considered for this study, based on the ranges defined by Reinert and Allen (2002): null-low, abuse, and risk of dependence.

#### Additional data

Demographic, clinical, and social/family variables related to gambling were measured using a semi-structured, face-to-face clinical interview (Jiménez-Murcia et al., 2006).

### Procedure

The present study was carried out in accordance with the latest version of the Declaration of Helsinki. The Bellvitge University Hospital Ethics Committee of Clinical Research approved the study. Signed, informed consent was obtained from all participants.

### Statistical analyses

Statistical analysis was carried out with Stata13.1 for Windows (StataCorp., 2013). Comparison between categorical variables was done using chi-square (χ^2^) tests and between quantitative variables through analysis of variance (ANOVA). Bonferroni-Finner's correction controlled the inflation in Type-I error due to multiple statistical comparisons. Effect sizes for the proportion and mean comparisons were estimated through Cohen's-*d* coefficient, considering |*d*| > 0.50 as a moderate effect size and |*d*| > 0.80, a high effect size.

## Results

### Epidemiology for the comorbidity CBB+GD

Considering the initial sample of all patients who arrived to the Unit during the time of data recruitment (*n* = 3363), the prevalence of patients who met criteria for only-GD was 92.0% (95%CI: 91.0–92.9%), for only-CBB, 3.06% (95%CI: 2.53–3.70%), and for comorbid CBB+GD 0.71% (95%CI: 0.48–1.06%). The effect size of the comorbidity of CBB+GD was OR = 5.2 (95%CI: 3.2–8.3).

**Figure 1:**
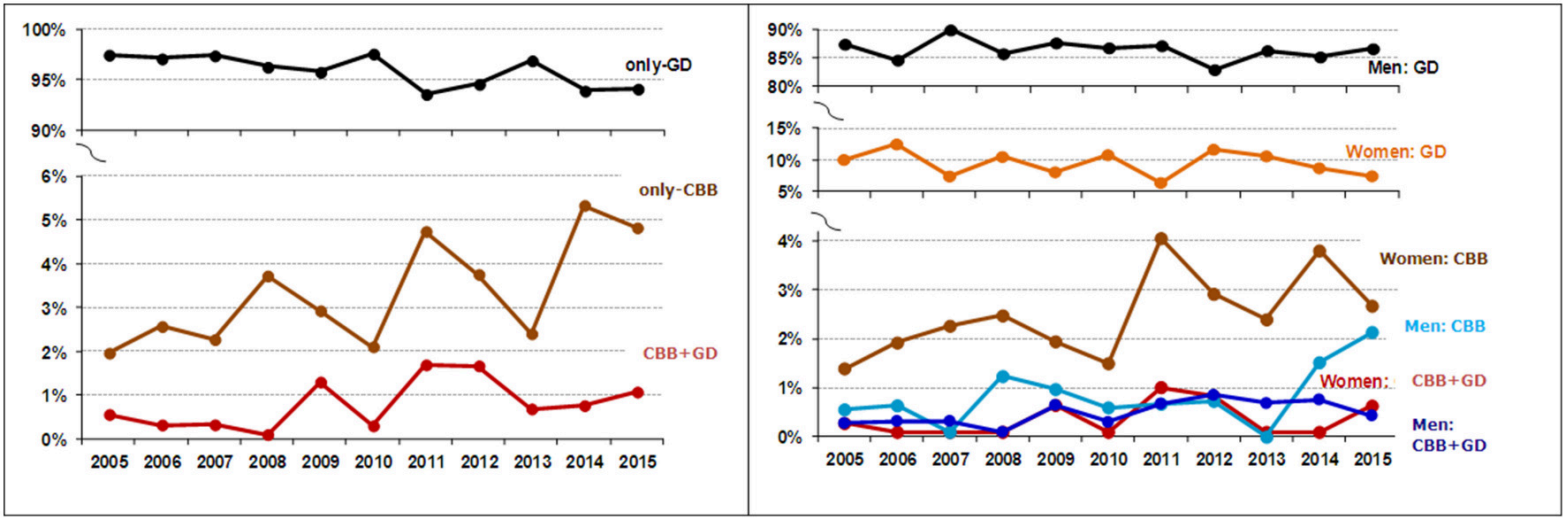
**Evolution of the consultation prevalence**. CBB, compulsive buying behavior; GD, gambling disorder.

In the final study sample (*n* = 3221), estimated prevalences were quite similar. The highest prevalence was for the diagnostic condition only-GD (96.1%; 95%CI: 95.3–96.7%), followed by only-CBB (3.20%; 95%CI: 2.64–3.86%) and comorbid CBB+GD (0.75%; 95%CI: 0.50–1.11%). Considering the subsample of patients who presented GD (*n* = 3118), the presence of the comorbidity CBB achieved a relative low point prevalence (0.77%; 95%CI: 0.52–1.14%). However, while considering the subsample of CBB patients (*n* = 127), the estimated prevalence for comorbid GD was relatively high (18.9%; 95%CI: 13.0–26.6%). Figure 1 shows the prevalence of patients attending the unit for treatment due to only-CBB, only-GD and CBB+GD during the time of data recruitment (2005–2015), for the total sample stratified by the patients' sex (separately for men and women).

### Comparison between only-CBB, only-GD and CBB+GD

Table 1 shows the comparison of the categorical variables of the study: sex, origin, education-level, civil status, employment status and substances use-abuse. Compared to the other diagnostic subtypes (CBB and GD), the comorbid condition CBB+GD was characterized by differences in the distributions of patients' gender, civil status, and the presence of other addictive behaviors (sex, videogame, and internet): CBB+GD included a lower proportion of single patients and a higher proportion of divorced/separated patients. These patients were also characterized by a higher proportion of women compared to only-GD, a higher proportion of men compared to only-CBB, and higher risk of other behavioral addictions compared to only-GD. Other differences were found when comparing only-CBB to only-GD; lower education levels, higher prevalence of tobacco, alcohol and other drug use, and a lower risk of internet addiction were related to the presence of only-GD.

**Table 1:** **Comparison of categorical variables between groups**.

	**Proportion (%)**			**Group Chi-square tests**			**CBB**+**GD vs. Only-CBB**		**CBB**+**GD vs. Only-GD**		**Only-CBB vs. Only-GD**	
	**Only-CBB**	**Only-GD**	**CBB+GD**									
	***n* = 103 (%)**	***n* = 3094 (%)**	***n* = 24 (%)**	**χ^2^**	***df***	***p***	***p***	***|d|***	***p***	***|d|***	***p***	***|d|***
Sex: Female	75.7	10.0	37.5	415.1	2	<0.001^*^	<0.001^*^	**0.84**	<0.001^*^	**0.68**^**†**^	<0.001^*^	**1.78**^**†**^
Origin: Immigrant	1.9	6.4	4.2	3.60	2	0.207	0.518	0.13	0.652	0.10	0.065	0.23
Education: Primary	35.1	57.6	47.8	51.20	4	<0.001^*^	0.431	0.26	0.313	0.20	<0.001^*^	0.46
Secondary	42.3	36.4	39.1					0.06		0.06		0.12
University	22.7	6.0	13.0					0.25		0.24		**0.51**^**†**^
Civil status: Single	37.0	35.8	17.4	9.56	4	0.048^*^	0.047^*^	**0.50**^**†**^	0.009^*^	**0.50**^**†**^	0.885	0.03
Married-couple	48.0	50.4	47.8					0.00		0.05		0.05
Divorced	15.0	13.8	34.8					**0.50**^**†**^		**0.51**^**†**^		0.03
Employed: Yes	49.5	56.4	45.8	2.90	2	0.235	0.746	0.07	0.300	0.21	0.172	0.14
Substances use												
Smoker: Yes	36.9	61.2	45.8	26.68	2	<0.001^*^	0.418	0.18	0.124	0.31	<0.001^*^	**0.50**^**†**^
AUDIT: Low	96.1	85.1	87.5	9.87	4	0.043^*^	0.099	0.32	0.889	0.07	0.008^*^	0.38
Abuse	3.9	14.2	12.5					0.32		0.05		0.36
Risk dependence	0.0	0.7	0.0							0.12		0.12
Other drugs: Yes	3.0	9.1	4.2	6.70	2	0.043^*^	0.765	0.06	0.403	0.20	0.034^*^	0.26
Other addictions: ^a^Any other												
	5.8	1.5	8.3	18.45	2	<0.001^*^	0.649	0.23	0.006^*^	0.10	<0.001*	0.32
Sex: Yes	1.0	0.1	4.2	30.02	2	<0.001^*^	0.257	0.20	<0.001^*^	0.28	0.123	0.12
Gaming: Yes	1.9	0.6	4.2	6.43	2	0.040^*^	0.518	0.13	0.036^*^	0.23	0.157	0.11
Internet: Yes	3.9	1.0	8.3	19.05	2	<0.001^*^	0.355	0.19	<0.001^*^	0.36	**0.022**	0.19

*CBB, compulsive buying behavior; GD, gambling disorder*.

a *Includes sex or videogame or internet addiction*.

* *Bold: significant comparison (0.05 level)*.

† *Bold: effect size into the range moderate (|d| > 0.50) to high (|d| > 0.80)*.

*p-values include Bonferroni-Finner correction*.

Table 2 shows the comparison of the quantitative variables of the study: patients' age, the age of onset and duration of the behavioral addiction, income (both patient and overall family income), gambling levels (SOGS and DSM-IV questionnaire total scores), general psychopathology levels (SCL-90-R scale scores) and personality traits (TCI-R scale scores). The co-occurrence of CBB+GD was characterized by older age and age of disorder onset, and a shorter duration of the addictive behavior. No further differences emerged when comparing comorbid CBB+GD vs. only-CBB. However, compared to only-GD, the simultaneous presence of CBB+GD was related to higher psychopathology (higher mean symptoms in many SCL-90-R scales) and higher scores in the personality trait harm avoidance. Remaining differences were found comparing only-CBB and only-GD; the presence of compulsive buying was related to higher psychopathology symptom levels (in all the SCL-90-R scales) and higher mean scores in the personality domains novelty seeking, harm avoidance and reward dependence.

**Table 2:** **Comparison of quantitative variables between groups**.

		**Means**			**Group ANOVA**		**CBB**+**GD vs. Only-CBB**		**CBB**+**GD vs. Only-GD**		**Only-CBB vs. Only-GD**	
		**Only-CBB**	**Only-GD**	**CBB+GD**								
		***n = 103***	***n = 3094***	***n = 24***	***F*_(2, 3218)_**	***p***	***p***	***|d|***	***p***	***|d|***	***p***	***|d|***
Age (years)		42.57	42.76	49.83	3.31	0.050^*^	0.017^*^	**0.60**^**†**^	0.010^*^	**0.53**^**†**^	0.888	0.02
Onset (years)		38.06	38.12	46.78	4.72	0.027^*^	0.005^*^	**0.70**^**†**^	0.002^*^	**0.64**^**†**^	0.965	0.00
Duration (years)		4.50	4.94	2.47	2.15	0.116	0.146	0.45	0.050^*^	**0.50**^**†**^	0.474	0.08
Patient monthly income (€)		1194	1197	1349	0.48	<0.001^*^	0.368	0.18	0.326	0.19	0.969	0.00
Family monthly income (€)		2365	2048	1945	2.68	<0.001^*^	0.136	0.43	0.677	0.10	0.023^*^	0.29
Cumulate debts (€)		12882	5652	9938	16.05	<0.001^*^	0.325	0.15	0.113	0.28	<0.001^*^	**0.50**^**†**^
SOGS: total score	α = 0.76	—	10.02	9.08	1.93	0.165	—	—	0.165	0.25	—	—
DSM-IV Stinchfiled's questionnaire	α = 0.81	—	6.73	5.92	3.78	0.052	—	—	0.052	0.32	—	—
SCL-90-R: Somatization	α = 0.89	1.47	0.94	1.17	18.76	<0.001^*^	0.140	0.29	0.214	0.25	<0.001^*^	**0.55**^**†**^
SCL-90-R: Obs./comp	α = 0.86	1.81	1.12	1.49	32.50	<0.001^*^	0.109	0.30	0.048^*^	0.37	<0.001^*^	**0.73**^**†**^
SCL-90-R: Int. sensitivity	α = 0.85	1.48	1.03	1.46	15.54	<0.001^*^	0.926	0.02	0.020^*^	0.40	<0.001^*^	0.48
SCL-90-R: Depressive	α = 0.89	2.06	1.48	1.89	19.99	<0.001^*^	0.428	0.15	0.046^*^	0.37	<0.001^*^	**0.57**^**†**^
SCL-90-R: Anxiety	α = 0.87	1.51	1.00	1.36	18.58	<0.001^*^	0.469	0.14	0.049^*^	0.40	<0.001^*^	**0.52**^**†**^
SCL-90-R: Hostility	α = 0.82	1.28	0.91	1.15	9.86	<0.001^*^	0.520	0.13	0.187	0.27	<0.001^*^	0.40
SCL-90-R: Phobic	α = 0.80	0.87	0.49	0.78	15.30	<0.001^*^	0.563	0.09	0.063	0.31	<0.001^*^	0.45
SCL-90-R: Paranoid	α = 0.76	1.31	0.91	1.48	16.88	<0.001^*^	0.382	0.15	0.001^*^	**0.54**^**†**^	<0.001^*^	0.47
SCL-90-R: Psychotic	α = 0.84	1.11	0.89	1.25	5.58	0.004^*^	0.453	0.15	0.037^*^	0.42	0.008^*^	0.25
SCL-90-R: GSI	α = 0.98	1.51	1.04	1.43	21.62	<0.001^*^	0.641	0.09	0.016^*^	0.44	<0.001^*^	**0.58**^**†**^
SCL-90-R: PST	α = 0.98	54.83	46.20	50.57	7.48	0.001^*^	0.420	0.18	0.363	0.18	<0.001^*^	0.39
SCL-90-R: PSDI	α = 0.98	2.29	1.88	2.29	25.71	<0.001^*^	0.992	0.00	0.002^*^	**0.58**^**†**^	<0.001^*^	**0.64**^**†**^
TCI-R: Novelty seeking	α = 0.70	115.19	108.73	111.10	9.01	<0.001^*^	0.241	0.27	0.452	0.16	<0.001^*^	**0.50**^**†**^
TCI-R: Harm avoidance	α = 0.81	109.96	101.25	110.95	14.01	<0.001^*^	0.813	0.05	0.011^*^	**0.52**^**†**^	<0.001^*^	**0.51**^**†**^
TCI-R: Reward depend	α = 0.77	105.28	99.70	99.43	5.77	0.007^*^	0.116	0.35	0.937	0.02	0.001^*^	**0.50**^**†**^
TCI-R: Persistence	α = 0.87	107.86	109.34	108.86	0.21	0.809	0.847	0.05	0.919	0.02	0.519	0.07
TCI-R: Self-directed	α = 0.85	124.30	128.11	122.81	1.90	0.209	0.778	0.06	0.269	0.24	0.104	0.16
TCI-R: Cooperativen	α = 0.80	136.42	132.04	133.86	2.83	0.104	0.545	0.13	0.635	0.09	0.019^*^	0.26
TCI-R: Self-Trans	α = 0.83	66.04	64.31	67.38	0.94	0.438	0.722	0.08	0.365	0.18	0.296	0.11

*CBB, compulsive buying disorder; GD, gambling disorder*.

—* Not registered for the group*.

* *Bold: significant comparison (0.05 level)*.

† *Bold: effect size into the range moderate (|d|>0.50) to high (|d|>0.80)*.

*α: Cronbach's-alpha for the self-report scale in the sample. p-values include Bonferroni-Finner correction*.

**Figure 2:**
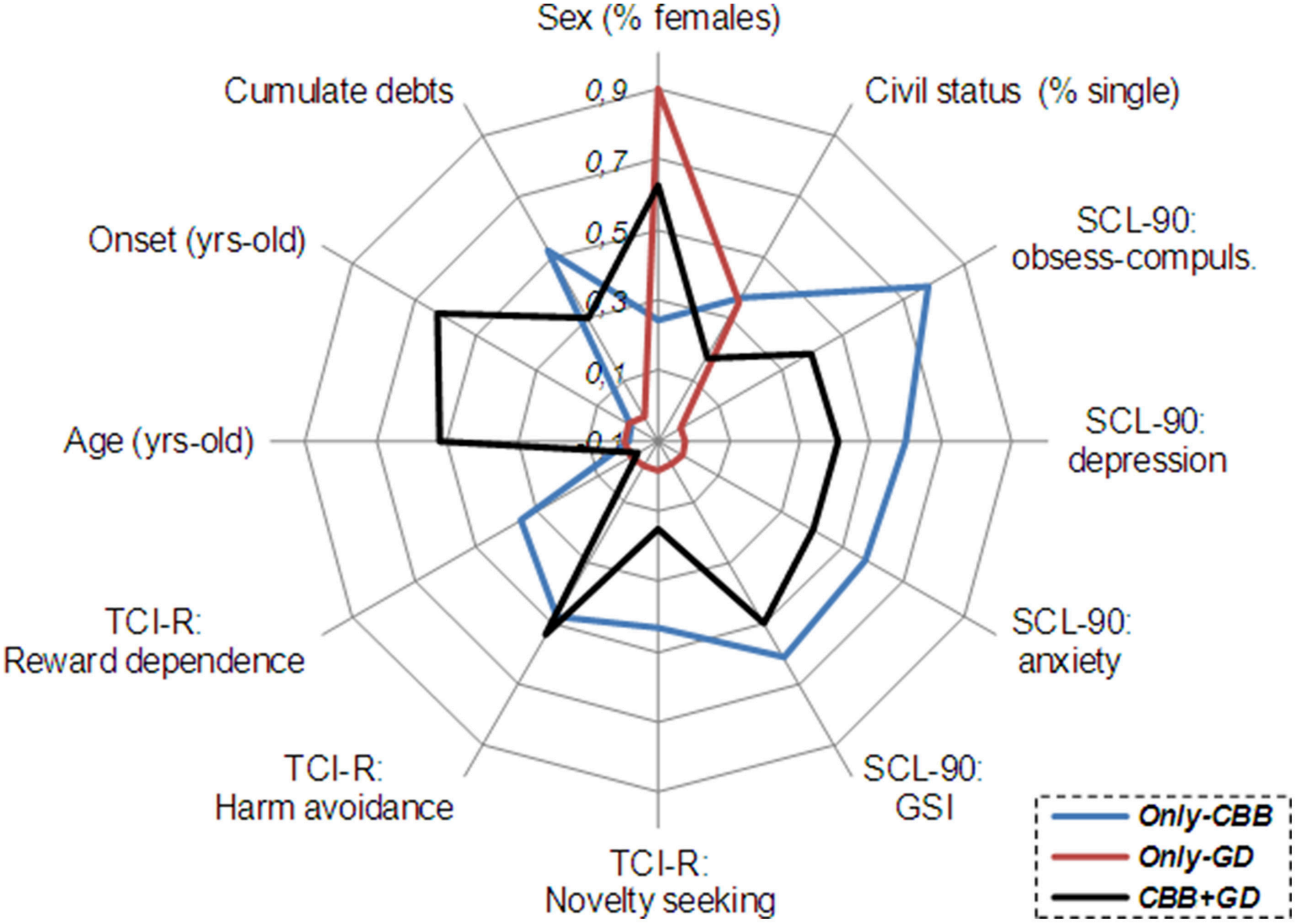
**Radiar-chart for the main clinical variables of the study**. CBB, compulsive buying behavior; GD, gambling disorder.

Figure 2 includes a radar-chart to graphically summarize the main clinical differences between comorbid CBB+GD and the other two study groups. The percentage of women was plotted for gender distribution and the percentage of single patients for civil status. The z-standardized mean scores in the sample were plotted for the quantitative clinical measures (standardization was carried out within different ranges—the minimum to maximum values—of these variables).

## Discussion

This study analyzes the frequency of the co-occurrence of CBB+GD, and the specific characteristics of this comorbidity compared to only-CBB and only-GD. Findings of this study show that CBB is not common in patients who met DSM criteria for GD (around 0.8%), but GD frequently occurs in patients who met criteria for CBB (around 19%). The psychiatric comorbid condition CBB+GD is characterized by a unique distribution of sociodemographic variables, a higher risk of the co-occurrence of other behavioral addictions (sex, gaming, or internet) and higher psychopathology (when compared to only-GD).

Results for gender in this study are consistent with those reported in the literature, which show that CBB and GD are strongly related to opposite sexes. Cross-sectional, community-based and clinical surveys suggest that patients who meet criteria for CBB are mainly women (between 80 and 95%; Fattore et al., 2014), while GD is found mainly in men (around 75%; Shin et al., 2014; Slutske et al., 2015). Suitable reasons as to why women are more likely to have CBB than men can be explained from a socio-cultural perspective (Granero et al., 2009). First, the stigmatization of social recognition of addictions such as gambling disorder or sexual addiction might be a protective factor for women as they may be less likely to engage in culturally sanctioned activities than men (Farré et al., 2015). In addition, women generally spend more time shopping than men do (Dittmar, 2005), which consequently increases the risk of exposure to this activity and make women more vulnerable to developing CBB (Dittmar et al., 2004).

Differences in age and the age of onset of CBB and GD are relevant in this study. Epidemiological evidence shows that the onset of GD varies widely between studies, but a common trend toward gambling at younger ages has emerged in many developed countries (Volberg et al., 2010; Ashton et al., 2014; Granero et al., 2014), mainly as a consequence of the expansion of opportunities for gambling, the increased use of new technology and the legalization of online gambling (Jiménez-Murcia et al., 2013; Gainsbury et al., 2015). It is understood that the emergence of gambling behavior at an early age is a powerful risk factor for the rapid development and evolution of GD (Johansson et al., 2009; Castrén et al., 2013). As a whole, epidemiological research suggest that other problematic behaviors (including CBB) usually become problematic in late adolescence and early adulthood (Balogh et al., 2013; Maraz et al., 2015b), since it is during this developmental state when impulsivity and risky behaviors may be most socially tolerated or even promoted by peers (Dayan et al., 2010; Hartston, 2012; Sussman and Arnett, 2014). However, in our study, patients' age and the age of onset of the problematic addictive behavior greatly differed between diagnostic subtypes, with only-CBB being the youngest (mean age was 42.6 and mean age of onset, 38.1 years) and CBB+GD being the oldest (mean age 49.8 and mean age of onset, 46.8). One possible explanation for this discrepancy is that the measure to determine “onset” in our study referred to the moment when the patients themselves recognized that their primary behavioral addiction had become uncontrollable and harmful. Therefore, it is likely that their buying or gambling behavior had previously become problematic without the subject having perceived it as being so. It also must be highlighted that some representative surveys in Europe over the last years have revealed increases in the estimated prevalence of behavioral addictions in adult populations, particularly CBB (Mueller et al., 2010a).

Evident differences in the main clinical measures of our study (symptom measures, cumulated debs, personality traits, and concurrence with other behavior addictions) also appeared when comparing the three diagnostic subtypes. The psychological profile for CBB+GD was very similar to that of only-CBB, and was clearly worse than the clinical profile for only-GD. This result outlines the potential existence of underlying factors specifically associated to the presence of CBB that should contribute to explain differences between this disorder and GD.

Finally, our results must be interpreted in light of their limitations. Possibly due to higher awareness of this condition, the number of only-GD patients in our sample was vastly higher than the number of only-CBB and CBB+GD patients, and the same can be said of the distribution of sexes across groups. Future research should include larger, more balanced samples so as to overcome these limitations. Qualitative information regarding the chronology of GD and CBB was also missing from our analyses. In cases of CBB+GD patients, the possibility that the consequences of one condition may influence the onset of another is a factor that should be considered in future studies. Also, the lack of consensus regarding the diagnostic criteria for CBB also limits the generalizability of our results is an issue that must be properly settled by the research community. Finally, we also wish to stress that the features of treatment-seeking patients from our Unit may not necessarily match those found in other community samples.

## Conclusions

In conclusion, the comorbidity CBB+GD is related to a specific phenotype which is particularly different to that in only-GD patients. These findings highlight the recognition that CBB and GD do not constitute homogenous groups and that CBB should be considered as an identifiable and distinct disorder. Further research is required to explore the underlying causes of the variability observed in CBB and GD profiles, particularly regarding the emotional and functional toll of the co-occurrence of CBB+GD. Preventive mental health and intervention services could benefit from screening and assessing for the comorbidity CBB+GD to provide treatment approaches that adequately manage the co-occurrence of these disorders. As GD and CBB are both behavioral addictions and are characterized by similar patterns of behavior, treatment strategies to address either condition could share many common features. However, specific treatment programs for patients who have comorbid GB and CBB may need to be developed. Such programs should focus on reducing overspending and gambling episodes via psychoeducation and behavioral interventions related to the main aspects of both disorders (e.g., stimulus control with money management strategies and relapse prevention with techniques that allow patients to identify high-risk situations).

## Author contributions

RG, FF, ST, JM, and SJ designed the experiment based on previous results and clinical experience of MB, AP, LM, NA, and MG. RG, FF, TS, GM, NM and SJ conducted the experiment, analyzed the data, and provided a first draft of the manuscript. VM further modified the manuscript. FF and SJ further modified the manuscript.

## Funding

This manuscript and research was supported by grants from Instituto de Salud Carlos III (FIS PI11/00210, FIS14/00290, CIBERObn, CIBERsam, and cofunded by FEDER funds/ European Regional Development Fund (ERDF) - a way to build Europe) and PROMOSAM (PSI2014-56303-REDT). CIBERObn and CIBERSAM are both an initiative of ISCIII.

### Conflict of interest statement

The authors declare that the research was conducted in the absence of any commercial or financial relationships that could be construed as a potential conflict of interest.
